# The effect of parental factors in children with large cup-to-disc ratios

**DOI:** 10.1371/journal.pone.0175900

**Published:** 2017-04-25

**Authors:** Hae-Young Lopilly Park, Min Ji Ha, Sun Young Shin

**Affiliations:** Department of Ophthalmology & Visual Science, Seoul St. Mary’s Hospital, College of Medicine, The Catholic University of Korea, Seoul, South Korea; Bascom Palmer Eye Institute, UNITED STATES

## Abstract

**Background:**

To investigate large cup-to-disc ratios (CDR) in children and to determine the relationship between parental CDR and clinical characteristics associated with glaucoma.

**Methods:**

Two hundred thirty six children aged 6 to 12 years with CDR ≥ 0.6 were enrolled in this study. Subjects were classified into two groups based on parental CDR: disc suspect children with disc suspect (CDR ≥0.6) parents and disc suspect children without disc suspect parents. Ocular variables were compared between the two groups.

**Results:**

Of the 236 disc suspect children, 100 (42.4%) had at least one disc suspect parent. Intraocular pressure (IOP) was higher in disc suspect children with disc suspect parents (16.52±2.66 mmHg) than in disc suspect children without disc suspect parents (14.38±2.30 mmHg, p = 0.023). In the group with disc suspect parents, vertical CDR significantly correlated with IOP (R = -0.325, p = 0.001), average retinal nerve fiber layer (RNFL) thickness (R = -0.319, p = 0.001), rim area (R = -0.740, p = 0.001), and cup volume (R = 0.499, p = 0.001). However, spherical equivalent (R = 0.333, p = 0.001), AL (R = -0.223, p = 0.009), and disc area (R = 0.325, p = 0.001) significantly correlated with vertical CDR in disc suspect children without disc suspect parents, in contrast to those with disc suspect parents. Larger vertical CDR was associated with the presence of disc suspect parents (p = 0.001), larger disc area (p = 0.001), thinner rim area (p = 0.001), larger average CDR (p = 0.001), and larger cup volume (p = 0.021).

**Conclusions and relevance:**

Family history of large CDR was a significant factor associated with large vertical CDR in children. In children with disc suspect parents, there were significant correlations between IOP and average RNFL thickness and vertical CDR.

## Introduction

In adults, large optic disc cups are often a sign of glaucoma. However, children show a lower prevalence of glaucoma than adults,[1] and there is larger proportion of non-glaucomatous cupping in children.[2–5] Furthermore, reliable and reproducible examinations of visual fields and intraocular pressures are difficult to obtain in pediatric patients,[6, 7] and thus in clinical practice, children with large optic disc cups often present a clinical dilemma.

Advances in ocular imaging technology provide a potential means of obtaining quantitative optic nerve head (ONH) values and retinal nerve fiber layer (RNFL) thickness measurements. Optical coherence tomography (OCT) measurements of the peripapillary RNFL have been shown to have good sensitivity to detect glaucoma.[8] Since both RNFL thickness and ONH parameters by OCT can be performed noninvasively and easily in children,[9, 10] several groups have reported descriptive studies of using both techniques in normal pediatric populations.[11–13] Despite the availability of modern imaging devices, the etiology of large optic disc cups in children remains uncertain.

Little information is known about children with large cup-to-disc ratios (CDR). Megalopapilla is a congenitally anomalous optic disc enlargement with a surface area greater than 2.5 mm^2^, originally described by Franceschetti & Bock.[14] Some have considered large discs with large cups in children physiologic.[15, 16] However, a previous study found that 13% of eyes with large cup-to-disc ratios progress to definite glaucoma over three years.[17] However, no study has been performed to determine whether large optic discs in children similarly progress to glaucomatous discs over time. Disc size and cup area have been shown to have a genetic heritability rate of up to 66%.[18, 19] Additionally, family history is a well-known risk factor for glaucoma development.[20] Understanding the clinical significance of large CDR in children from the clinical presentation of their parents regarding glaucoma could help guide treatment for children with large CDR.

Therefore, we investigated clinical characteristics in children with large CDR as they relate to parental CDR. We investigated the CDR of parents and examined how parental disc variables correlate with glaucoma clinical characteristics in their children.

## Methods

This study was a case-control cross-sectional study which was reviewed and approved by the Institutional Review Board of Seoul St. Mary's Hospital, Seoul, South Korea. The study adhered the principles of the Declaration of Helsinki. Written informed consent forms were obtained from the parents of the children. Two hundred thirty-six children with CDR ≥0.6 aged 6 to 12 years were enrolled from Seoul St. Mary's Hospital between 2010 and 2013.

All children underwent full ophthalmologic examinations, including best corrected visual acuity, cycloplegic refraction, slit-lamp examination, alternate cover test, intraocular pressure (IOP) measurements using Goldmann applanation tonometry, fundus and optic disc photographs, Spectral domain OCT (Cirrus HD-OCT, Carl Zeiss, Jena, Germany), and axial length (AL) from the IOL master (IOL master 500, Carl Zeiss, Jena, Germany). Optic disc photographs were taken using a nonmydriatic fundus camera (Topcon, Tokyo, Japan) with a digital camera. Spherical equivalent (SE) was measured under cycloplegic refraction.

Inclusion criteria was children aged between 6 to 12 years with average CDR ≥0.6. Two specialists were enrolled to determine the CDRs using the disc photograph. The graders visually estimated the vertical and horizontal CDRs on the basis of the contour of the cup. The mean of vertical and horizontal CDR from the two observers was used, and CDR measurements that differed >0.2 between the two observers were not included. Exclusion criteria included the following: best corrected visual acuity worse than 20/25; IOP ≥21mmHg; pathological disc and cup, such as morning glory disc syndrome, optic nerve hypoplasia, or tilted disc syndrome; abnormal findings on fundus and disc photography; family history of glaucoma; abnormal prenatal history; abnormal developmental history; and non-cooperation from children.

Parental CDR was measured, and subjects were classified into two groups based on parental CDR: disc suspect children with disc suspect (CDR ≥0.6) parents and disc suspect children without disc suspect parents. Ocular variables were compared between the two groups. Either father or mother having CDR ≥0.6 was included and analyzed. If both parents had CDR ≥0.6, only one parent was selected randomly.

Patients were scanned with Cirrus OCT (software version 5.0.1, Carl Zeiss Meditec). OCT scans were performed with FAST RNFL thickness protocols using internal fixation. The optic disc cube is a glaucoma scan protocol that images the optic disc and the parapapillary retinal region covering an area of 6 × 6 mm^2^ (200 × 200 data points). The built-in algorithms locate the center of the optic disc even if it is not well center in the scan image. The software identifies the disc center by finding a dark spot near the center of the scan that has a shape and size consistent with a range of optic discs. Five ONH parameters are measured: rim area, disc area, average CDR, vertical CDR, and cup volume. The average CDR is given by the square-root of the ratio of the area of the cup to the area of the disc. The vertical CDR is the ratio of the cup diameter to the disc diameter in the vertical meridian. Cup volume is a 3-dimensional measurement defined as the volume between a plane created by the cup outline at the vitreous interface and the posterior surface of the ONH. Only data with a signal strength greater than 6 without any motion artifacts was used.

Statistical analyses were performed using commercial software (SPSS ver. 17.0; SPSS Inc, Chicago, IL). For statistical evaluation, only one eye, randomly chosen, was considered for each patient. Student’s *t*-test was used to compare data between children with large CDRs versus controls. Chi-square test was used to analyze categorical variables. Possible relationships between peripapillary RNFL thickness and ONH parameters were analyzed using the Pearson correlation. To validate the agreement between graders to identify the presence of CDR ≥0.6, Kappa statistics was performed, and scores ≥ 0.75, between 0.40 and 0.75, and ≤ 0.4 were considered to be excellent, moderate, and poor, respectively.[21] A p value < 0.05 was considered statistically significant.

## Results

Of the 236 disc suspect children enrolled in this study, 100 (42.4%) had at least one disc suspect parent. The identification of disc suspects with the definition of CDR ≥0.6 between graders showed excellent agreement (κ = 86.7). Among 100 children with enlarged CDR, 58 (58%) had disc suspect father, 30 (30%) had disc suspect mother, and 12 (12%) had disc suspect of both parents. We compared the baseline characteristics of disc suspect children with and without disc suspect parents (Table 1). Children without disc suspect parents were older (8.07±4.43 years) than those with disc suspect parents (6.80±2.57 years, p = 0.005). Sex ratio, SE, and AL were not different between the two groups. Intraocular pressure was significantly higher in children with disc suspect parents (16.52±2.66 mmHg) than in those without disc suspect parents (14.38±2.30 mmHg, p = 0.023). Rim area was smaller (1.29±0.29 mm^2^ compared to 1.35±0.37 mm^2^); however, the average (0.73±0.16) and vertical CDR (0.66±0.07) were larger in children with disc suspect parents than those without disc suspect parents (0.61±0.16 and 0.63±0.11, respectively).

**Table 1:** Baseline characteristics.

	Disc suspect children with disc suspect parents (n = 100)	Disc suspect children without disc suspect parents (n = 136)	*P* Value
**Age, year** **Gender, Male:Female** **Sperical equivalent, diopter** **Axial length, mm** **Intraocular pressure, mmHg** **Average RNFL thickness, μm** **Rim area, mm^2^** **Disc area, mm^2^** **Average CDR** **Vertical CDR** **Cup volume, mm^3^**	**6.80 ± 2.57** 60:40 -0.98 ± 2.05 23.45 ± 1.06 **16.52 ± 2.66** 99.95 ± 10.37 **1.29 ± 0.29** 2.58 ± 0.48 **0.73 ± 0.16** **0.66 ± 0.07** 0.36 ± 0.20	**8.07 ± 4.43** 66:70 -0.91 ± 2.68 23.09 ± 1.14 **14.38 ± 2.30** 102.32 ± 10.03 **1.35 ± 0.37** 2.56 ± 0.53 **0.61 ± 0.16** **0.63 ± 0.11** 0.33 ± 0.25	0.005* 0.081^†^ 0.129* 0.406* 0.023* 0.663* 0.012* 0.235* 0.001* 0.001* 0.514*

Data are presented as mean ± standard deviation.

RNFL = retinal nerve fiber layer; CDR = cup-to-disc ratio.

*Independent t-test.

^†^Chi-square test.

Correlations in ocular and ONH parameters between the disc suspect children with disc suspect parents and their parent were analyzed. There were significant correlations between IOP (R = 0.774, p = 0.001), AL (R = 0.445, p = 0.003), rim area (R = 0.493, p = 0.001), and vertical CDR (r = 0.376, p = 0.044; Table 2).

**Table 2:** Correlation of ocular parameters between disc suspect children and their disc suspect parents.

Correlation of parameters between child and parent	*R*	*P* Value*
IOP Sperical equivalent Axial length Average RNFL thickness Rim area Disc area Average CDR Vertical CDR Cup volume	**0.774** 0.082 **0.445** 0.275 **0.493** 0.006 0.009 **0.376** 0.058	**<0.001** 0.605 **0.003** 0.098 **0.001** 0.969 0.954 **0.044** 0.717

IOP = intraocular pressure; RNFL = retinal nerve fiber layer; CDR = cup-to-disc ratio.

*R* = correlation coefficient.

*Pearson correlation analysis.

**Fig 1:**
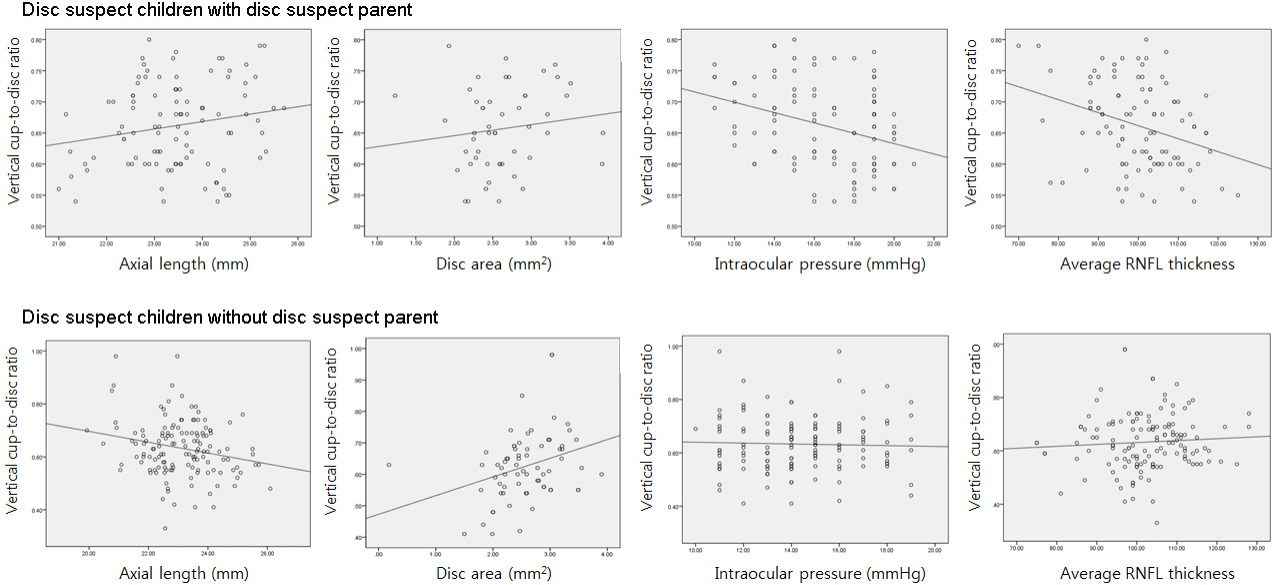
Scatterplot between ocular parameters and vertical cup-to-disc ratio in disc suspect children with or without disc suspect parents. Vertical cup-to-disc ratio showed significant correlation with intraocular pressure and average retinal nerve fiber layer thickness in disc suspect children with disc suspect parent.

In children with disc suspect parents, vertical CDR significantly correlated with IOP (R = -0.325, p = 0.001), average RNFL thickness (R = -0.319, p = 0.001), rim area (R = -0.740, p = 0.001), and cup volume (R = 0.499, p = 0.001). However, SE (R = 0.333, p = 0.001), AL (R = -0.223, p = 0.009), and disc area (R = 0.325, p = 0.001) showed a significant correlation with vertical CDR in disc suspect children without disc suspect parents; this was in contrast to disc suspect children with disc suspect parents (Table 3). As shown in Fig 1, those with disc suspect parents showed a significant negative correlation between IOP and vertical CDR and between average RNFL thickness and vertical CDR. In contrast, those without disc suspect parents showed a significant correlation between AL and vertical CDR and between disc area and vertical CDR.

**Table 3:** Correlation between ocular parameters and cup-to-disc ratio in disc suspect children with or without disc suspect parents.

	Disc suspect children with disc suspect parents				Disc suspect children without disc suspect parents			
	Average CDR		Vertical CDR		Average CDR		Vertical CDR	
	*R*	*P* Value*	*R*	*P* Value*	*R*	*P* Value*	*R*	*P* Value*
**Age** **IOP** **Sperical equivalent** **Axial length** **Average RNFL thickness** **Rim area** **Disc area** **Cup volume**	0.144 **-0.230** **-0.278** 0.168 -0.134 **-0.313** -0.172 **0.314**	0.153 **0.021** **0.005** 0.095 0.183 **0.002** 0.086 **0.001**	0.055 **-0.325** -0.018 0.187 **-0.319** **-0.740** 0.119 **0.499**	0.589 **0.001** 0.857 0.062 **0.001** **<0.001** 0.236 **<0.001**	0.092 -0.160 0.141 -0.003 0.136 **-0.624** 0.123 **0.361**	0.288 0.063 0.102 0.972 0.114 **<0.001** 0.155 **<0.001**	0.013 -0.035 **0.333** **-0.223** 0.068 **-0.693** **0.325** **0.590**	0.883 0.689 **<0.001** **0.009** 0.430 **<0.001** **<0.001** **<0.001**

IOP = intraocular pressure; RNFL = retinal nerve fiber layer; CDR = cup-to-disc ratio.

*R* = correlation coefficient.

*Pearson correlation analysis.

To determine factors related to vertical CDR, regression analyses were performed. Larger vertical CDR was associated with the presence of disc suspect parents (p = 0.001), larger disc area (p = 0.001), thinner rim area (p = 0.001), larger average CDR (p = 0.001), and larger cup volume (p = 0.021) in multivariate analysis (Table 4).

**Table 4:** Regression analysis of associated factors with the vertical cup-to-disc ratio in disc suspect children.

Vertical CDR	Univariate analysis			Multivariate analysis		
	β	95% CI	*P* Value	β	95% CI	*P* Value
Age	0.001	-0.004 to 0.003	0.714			
Family history of enlarged CDR	0.029	0.005 to 0.053	0.017	0.025	0.011 to 0.040	0.001
Intraocular pressure	-0.002	-0.006 to 0.002	0.369			
Refraction	0.009	0.004 to 0.013	<0.001	0.003	-0.004 to 0.006	0.121
Axial length	-0.006	-0.016 to 0.005	0.284			
Average RNFL thickness	0.001	-0.002 to 0.002	0.256			
Disc area	0.047	0.025 to 0.070	<0.001	0.076	0.061 to 0.090	<0.001
Rim area	-0.187	-0.212 to -0.163	<0.001	-0.202	-0.224 to -0.181	<0.001
Average CDR	0.335	0.281 to 0.388	<0.001	0.147	0.106 to 0.187	<0.001
Cup volume	0.221	0.179 to 0.262	<0.001	0.039	0.006 to 0.071	0.021

RNFL = retinal nerve fiber layer; CDR = cup-to-disc ratio; CI = confidence intervals.

## Discussion

In the current study, we observed that 42.3% of children with large cupping had at least one disc suspect parent. A family history of large CDR was a significant factor associated with large vertical CDR in children. All children were included on the basis of CDR larger than 0.6; however, baseline characteristics in terms of IOP and CDR significantly varied depending on parental CDR. Importantly, children with large cupping who had parents with large cupping showed a higher IOP and greater CDR when compared to children without parents with large cupping. Additionally, analysis of the relationship between ocular and ONH characteristics showed that parental factors affect this relationship. Children with disc suspect parents showed a significant correlation between IOP and average RNFL thickness with vertical CDR. However, in children without disc suspect parents, there was significant correlation between SE and AL with vertical CDR.

A previous study reported that additive genetic factors can explain variations in CDR in a twin study.[19] However until now, no study has investigated the incidence of parental large cupping in children with large cupping. Recently, some studies have shown that disc size and cup area can be largely heritable, up to 66%.[18] Our study adds to this work, demonstrating that a large CDR may also be a heritable factor. Among related factors, a family history of large CDR in parents was significantly related to the degree of vertical CDR in children.

Most importantly, there have been no evidence-based guidelines on how children with large cupping should be managed. In addition, children are generally not as cooperative as adults and cannot undergo detailed ocular examinations such as the Humphrey VF test. One may not, however, exclude the possibility that eyes with large cupping may eventually develop a glaucoma-like optic neuropathy, as shown in a previous article on a 3-year-old boy with a congenital megalopapilla who developed rim loss, visual field loss, and visual field defects 10 years later. Our data suggest that performing ONH examinations in parents of the children with large CDR may help guide the management of these children.

In the present study, children with large CDR who had parents with large CDR showed higher IOP at a younger age. Rim area was smaller and cup parameters were all significantly larger in these children compared to those without large parental CDR. The CDR of children without large parental CDR seems to be related to myopic changes, since it was significantly correlated with SE, AL, and disc area. Disc tilting and peripapillary changes during myopic eyeball growth are known to affect the morphology of the ONH, including cupping of the optic disc. However, the CDR of children with larger parental CDR was significantly related to IOP and RNFL thickness, which are risk factors of glaucoma. Therefore, children with large CDR who also have parents with large CDR should be monitored for changes in IOP and RNFL thickness over time.

Our data was not consistent over average and vertical CDR. There were significant difference in both average and vertical CDR between disc suspect children with and without disc suspect parents. However, among all CDR parameters, only vertical CDR was significantly correlated between disc suspect children and disc suspect parents. And so, we did the further analysis using vertical CDR. Normally, the disc is vertically oval shaped and the cup is horizontally oval shaped. Therefore, changes in the vertical CDR is more prominent and consistent than the horizontal CDR. Previous studies show that vertical CDR is related to axial length, disc size, glaucomatous changes, gender, and race, which is variably reported with horizontal CDR.[22–25] Some studies investigating disc parameters only used vertical CDR for this reason.[26, 27] Average CDR was defined as the mean of vertical and horizontal CDR in this study. Since average CDR includes horizontal CDR which is not variable in terms of other ocular parameters, this could be the reason why our data only showed significance with vertical CDR, and not with average CDR in some analyses. Additionally, the significant correlation between parental vertical CDR and children vertical CDR, but no correlated between parental average CDR and children average CDR may show that parental factor influences vertical CDR, not the horizontal CDR. Further investigation may be needed to confirm our findings.

Several studies have reported on comparisons between megalopapilla patients and healthy controls or glaucoma patients.[10, 28] Megalopapilla is a congenitally anomalous optic disc enlargement with a surface area greater than 2.5 mm^2^. In children with parents with large CDR, vertical CDR was not related to disc area, supporting the idea that large CDR in these children should not be regarded as megalopapilla and physiologic large cupping in our study. Previous studies have reported that eyes with megalopapilla have larger optic disc diameter, disc and cup area, cup-to-disc and cup-to-disc area ratios than normal controls using SD-OCT in children.[28] However, rim area and rim volume are similar to those of normal controls, as is pRNFL thickness measured with SD-OCT. The average pRNFL thickness in our subjects was lower (99.95 and 102.32 μm) than this previous study (117.34 μm), which may be attributed to differences in the study population.

Our study had several limitations that must be acknowledged. First, healthy control children without enlarge CDR and their parental data is warranted to confirm heritability. However, only parents of children with enlarged CDR was evaluated. Our finding may need further investigations to confirm the relationships. Second, parental factor may be influenced by environmental and genetic factors. As our study was conduction in a country with a single ethnicity, the findings may be unique to this population, and may not be applicable to other populations. The findings could be different for other population with difference races or environmental conditions. At last, determination of CDR from disc photographs may be inaccurate, especially in children, since movement during photograph acquisition may affect centration of the optic disc, and also the quality of the photographs. Clinical measurement of CDR may be less accurate compared to other methods, such as optic disc analyzers. However, kappa statistic showed excellent agreement in determining CDR between graders.

In children, the finding of a large CDR despite a normal IOP usually prompts further evaluation and long term clinical monitoring. This is due to the possibility that large CDR may either be a sign of glaucomatous cupping or that the large CDR may not be physiologic cupping. An ONH examination of these patients’ parents could provide some clues on how these children should be managed. Studies evaluating the actual development of glaucoma in children with large CDR—especially according to their parental CDR—may provide further evidence for guiding this clinical decision.
